# Abortion-related emergency department visits in the United States: An analysis of a national emergency department sample

**DOI:** 10.1186/s12916-018-1072-0

**Published:** 2018-06-14

**Authors:** Ushma D. Upadhyay, Nicole E. Johns, Rebecca Barron, Alice F. Cartwright, Chantal Tapé, Alyssa Mierjeski, Alyson J. McGregor

**Affiliations:** 1 Department of Obstetrics, Gynecology & Reproductive Sciences, University of California, San Francisco, 1330 Broadway, Suite 1100, Oakland, California 94612 USA; 2 Department of Emergency Medicine, Warren Alpert Medical School of Brown University, 593 Eddy Street, Providence, RI 02903 USA

**Keywords:** Abortion, Emergency department, Emergencies, Hospital admission, Complications, Health policies

## Abstract

**Background:**

Media depictions and laws passed in state legislatures regulating abortion suggest abortion-related medical emergencies are common. An accurate understanding of abortion-related emergencies is important for informing policy and practice. We assessed the incidence of abortion-related emergency department (ED) visits in the United States (U.S.).

**Methods:**

We used a retrospective observational study design using 2009–2013 data from the Nationwide Emergency Department Sample, a nationally representative sample of U.S. ED visits from 947 to 964 hospitals across the U.S. per year. All ED visits among women of reproductive age (15–49) were included. We categorized ED visits by abortion relatedness and treatments received, and assessed whether the visit was for a major incident (defined as requiring blood transfusion, surgery, or overnight inpatient stay). We estimated the proportion of visits that were abortion-related and described the characteristics of patients making these visits, the diagnoses and subsequent treatments received by these patients, the sociodemographic and hospital characteristics associated with the incidents and observation care only (defined as receiving no treatments), and the rate of major incidents for all abortion patients in the U.S.

**Results:**

Among all ED visits by women aged 15–49 (189,480,685), 0.01% (*n* = 27,941) were abortion-related. Of these visits, 51% (95% confidence interval, 95% CI 49.3–51.9%) of the women received observation care only. A total of 20% (95% CI 19.3–21.3%) of abortion-related ED visits were for major incidents. One-fifth (22%, 95% CI 20.9–23.0%) of abortion-related visits resulted in admission to the same hospital for abortion-related reasons. Of the visits, 1.4% (*n* = 390, 95% CI 1.1–1.7%) were potentially due to attempts at self-induced abortion. In multivariable models, women using Medicaid (adjusted odds ratio, AOR 1.28, 95% CI 1.08–1.52) and women with a comorbid condition (AORs 2.47–4.63) had higher odds of having a major incident than women using private insurance and those without comorbid conditions. During the study period, 0.11% of all abortions in the U.S. resulted in major incidents as seen in EDs.

**Conclusions:**

Abortion-related ED visits comprise a small proportion of women’s ED visits. Many abortion-related ED visits may not be indicated or could have been managed at a less costly level of care. Given the low rate of major incidents, perceptions that abortion is unsafe are not based on evidence.

## Background

Emergency departments (EDs) are key sites of health-care delivery in the United States (U.S.), with 141.4 million visits in 2014 alone [1], and 55% of these visits are made by women. Although abortion-related complications are rare [2], the surge in legislation aimed at regulating abortion access [3] suggests that complications are common and that abortion is generally unsafe.

Since 2011, state legislatures across the U.S. have passed numerous laws that regulate abortion provision, many requiring abortion providers to obtain local hospital admitting privileges and have transfer agreements with nearby hospitals [3]. These laws are passed under a presumption that they are needed to protect women’s health and safety [4, 5] and that hospitalization as a result of abortion is an occurrence frequent enough to necessitate legislation formalizing the relationship between hospitals, abortion providers, and clinics.

National-level estimates of abortion-related ED visits do not exist. However, data from one state suggests that abortion-related ED visits are rare. In a study using data from California’s Medicaid program, Upadhyay et al. found that 0.03% of abortions were followed by an immediate ambulance transfer to an ED and 2.6% of abortions were followed by an abortion-related ED visit within 6 weeks of the abortion [2]. Another study that examined all medication abortions done by Planned Parenthood in 2009 and 2010 found an ED treatment rate of 0.10%, although medication abortions represented only about 23% of abortions at the time, and this study included only those ED visits that involved treatment [6]. In a study of outcomes of abortion procedures by family physicians in New York and Philadelphia, 0.3% of first-trimester medication and aspiration abortion patients were referred or went to an ED for assessment [7].

There is a paucity of published national data on the incidence and outcomes of ED visits after abortion. In this study, we examine the frequency of abortion-related ED visits, the frequency of major abortion-related incidents, and the characteristics of abortion-related ED visits in the U.S. using a nationally representative sample of ED visits.

## Methods

### Study design and data source

We conducted a retrospective observational study of abortion-related ED visits using data from the Nationwide Emergency Department Sample (NEDS), a nationally representative sample of ED visits. NEDS is a database of ED visits from 947 to 964 hospitals across the U.S. per year. Annually it includes more than 30 million unweighted visits, which represent more than 135 million weighted visits. NEDS was developed for the Healthcare Cost and Utilization Project (HCUP) and is maintained by the Agency for Healthcare Research and Quality. Data are available from 2006 onward. For this study, we utilized the five most recent years of data available (2009–2013). The study was certified exempt by the institutional review board of the University of California, San Francisco.

**Fig. 1:**
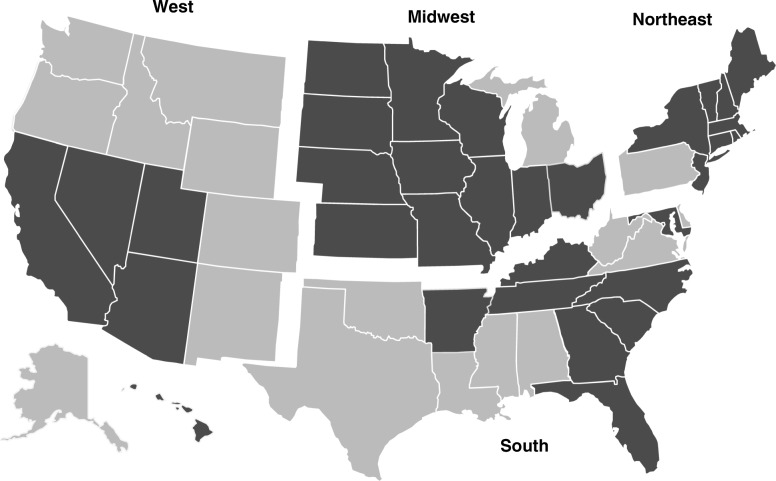
U.S. regions in the National Emergency Department Sample. States in darker shades contributed data to the National Emergency Department Sample during the study period, 2009–2013

Unweighted visits are data collected on actual visits, which are then weighted proportionately to the total number of ED visits in the country based on the sampling strategy. The NEDS is a stratified single-stage cluster sample of state-level ED data reported to HCUP. Using the American Hospital Association Annual Survey of Hospitals as the target universe, the available data are selected to approximate a 20% stratified sample of all U.S. hospital-based EDs. More details of the sampling of hospitals can be found on the HCUP website [8, 9]. The characteristics used for sample stratification in the NEDS are U.S. region, urban or rural location, teaching status, ownership, and trauma level (see Fig. 1 for region definitions and states contributing data).

The NEDS includes patient-level and hospital-level information. Each ED visit has patient-level demographic characteristics including age, sex, primary and secondary payment source, and zip code-based urbanicity and income quartile. Each ED visit also has clinical characteristics, including up to 15 diagnoses (International Classification of Diseases, 9th Revision [ICD-9] codes), up to 15 procedures or treatments (Healthcare Common Procedure Coding System [HCPCS] and Current Procedural Terminology [CPT] codes), injury codes, admission and discharge status, diagnosis and treatment codes for inpatient care if admitted to the same hospital, and total charges. Each visit also has the corresponding hospital code, and hospital characteristics such as region, trauma level, urban-rural location, and teaching status. In this dataset, 5 of 13 states in the West, 11 of 12 states in the Midwest, 8 of 16 states in the South, and 8 of 9 states in the Northeast were represented in the data. Midwest hospitals were represented the most. The trauma level of a hospital refers to how well equipped it is to provide care to patients with traumatic injuries. Trauma level influences patient composition and was key to sample stratification in the dataset. Hospital ownership was categorized by the data distributor according to information reported in the American Hospital Association Annual Survey Database. Ownership could be governmental, private non-profit, or private for-profit. Hospitals with religious affiliations, including Catholic hospitals, are included, but are not distinguished as such and may fall into private for-profit or private non-profit categories. Federal hospitals (Veterans Affairs and Department of Defense) were not included in the sample. Patient-level and hospital-level weights were also provided to generate nationally representative estimates. HCUP provided weights for the NEDS data and these were calculated at the hospital level after sampling by hospital strata. Patient weights were calculated first by stratifying the data by hospital characteristics (region, urbanicity, trauma level, teaching status, and ownership). Within each of these strata, a weight was generated by dividing the total number of ED visits in the U.S. in that year for that stratum (from American Hospital Association data) by the number of ED visits for that stratum in the NEDS data. Weighted data thus represent all ED visits in the U.S. for a year.

### Data preparation

**Fig. 2:**
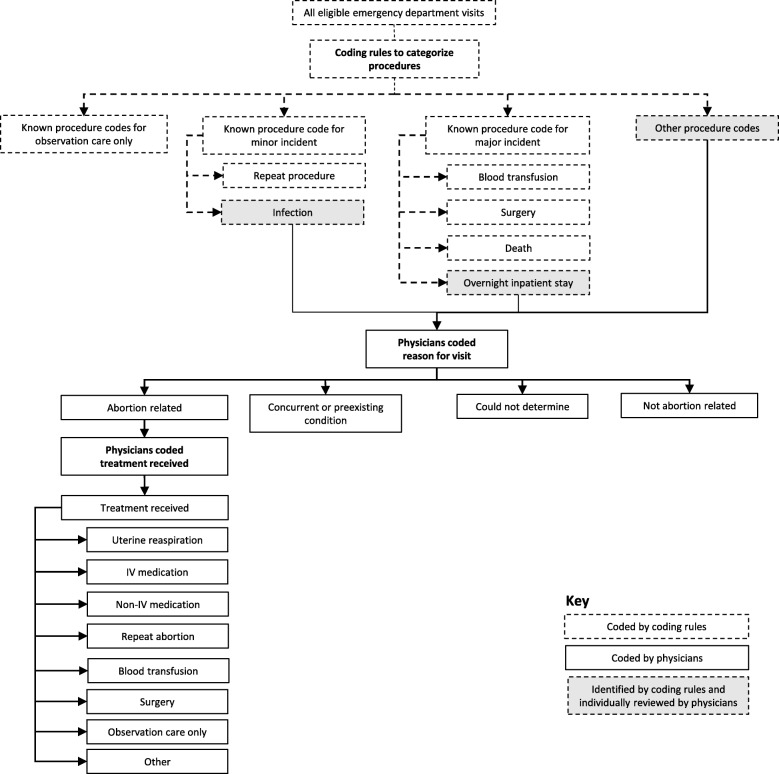
Flow chart for coding of abortion-related emergency department visits. IV intravenous

We identified all ED visits that had an ICD-9 diagnosis code for abortion (ICD-9 diagnosis codes 635, 636, 637, and 638). We categorized ED visits by abortion relatedness and treatments received, and assessed whether the visit was for a major or minor incident. Visit categorization was based on the previously developed Procedural Abortion Incident Reporting and Surveillance (PAIRS) Framework [10]. Based on procedure codes, visits that were for observation care, repeat procedures (codes present in the dataset were CPT/HCPCS procedure codes 59812, 59820, 59821, 59840, 59841, 59851, 59855, and 59856; and ICD-9 procedure codes 6901, 6902, 6909, 6951, 6952, 6959, 734, and 750), blood transfusions (CPT/HCPCS procedure codes 36430 and P9021; and ICD-9 procedure codes 9903–9905), and abortion-related surgeries (CPT/HCPCS procedure codes 49320, 58662, 58999, 59300, and 59898; and ICD-9 procedure codes 680, 6831, 6839, 6849, 6851, 6859, 6869, and 7491) were systematically coded without individual visit review. Where procedure and diagnosis codes for a visit did not fall into one of these categories, several authors who are emergency medicine physicians or students with physician supervision provided an individual review of each visit following a modified version of the PAIRS framework (see Fig. 2). After a joint review of 100 visits with refinement of the decision rules, the physicians reviewed the remaining uncategorized visits, resulting in 1642 reviewed ED visits in total.

For each reviewed visit, the emergency medicine physicians assigned the reason for the patient’s ED visit to one of five categories: abortion-related, concurrent condition, pre-existing condition, not abortion-related, or cannot determine. They classified an ED visit as abortion-related based on the constellation of diagnosis and procedure codes for that visit. Abortion-related visits included adverse events, such as hemorrhage or infection, and symptoms directly related to the procedure, such as abdominal pain and vomiting. Concurrent conditions were defined as conditions that may have been noticed during or exacerbated by abortion, but were not directly caused by the abortion, such as ovarian cysts, vaginitis, urinary tract infection, or anxiety/depression. Pre-existing conditions were defined as chronic conditions such as hypertension or diabetes. The data were also categorized with regards to treatments received for abortion-related complaints. Categories of treatment included uterine reaspiration (which involves suction, not an incision, and does not meet the criteria for surgery), intravenous (IV) and non-IV medication, repeat abortion, blood transfusion, surgery, observation care, or other. The medication category excluded codes for injections or infusion of a therapeutic substance if no accompanying medication was listed. The observation care category included women who had routine testing for their symptoms but did not receive any medications or other treatments. This included women who had IV fluids, blood work, testing for sexually transmitted infections, and diagnostic imaging studies, but no treatments. The NEDS dataset also included information on whether patients were admitted as an inpatient to the same hospital, discharged home, transferred to another facility, or left against medical advice. Among women who were admitted, the reason for visit and treatment information in their chart was used to determine if the admission was likely abortion-related.

Major and minor incidents were systematically coded, with major incidents defined as those requiring an overnight inpatient stay, blood transfusion, or surgery. Minor incidents were defined as all other incidents that involved an abortion-related diagnosis or treatment. All overnight stays were further reviewed by physicians, and a group of treatments for an abortion-related diagnosis that together required the patient to stay overnight qualified as a major incident. Visits that involved concurrent conditions, pre-existing conditions, or visits that were not abortion-related were categorized as no incident. We identified the prevalence of comorbid conditions based on diagnosis codes. These included three key conditions hypothesized to be associated with abortion-related adverse events: diabetes, hypertension, and having an overweight/obese body mass index (BMI) [11–14].

ED visits were additionally categorized as being potentially indicative of a self-induced abortion. The physician team individually reviewed all abortion-related visits that had diagnosis codes of illegal abortion, failed attempted abortion, and certain injury codes (including poisonings and indications of self-harm). They looked at all of the diagnosis codes for that case and made a clinical judgement based on the group of codes together and their ED experience. Visits that were coded as illegal abortions, particularly those that included injury codes, were more often considered potentially self-induced. In addition, visits that included injury codes consistent with self-induction were categorized as such. Cases which were unlikely to have been abortion-related were removed.

### Statistical analysis

We estimated the number of abortion-related ED visits annually in the U.S. and the proportion of ED visits among women of reproductive age (15–49) that were for abortion-related care. We examined the characteristics of the sample and compared these to published estimates of the characteristics of abortion patients [15]. We also described outcomes including treatments received and discharge status. Based on treatments received, ED visits were categorized as being for a major incident, a minor incident, no incident, or that the incident type could not be determined. We then built multivariable logistic regression models to examine the factors associated with major incidents and observation care, controlling for sociodemographic characteristics, comorbidities, and hospital characteristics with the ED visit as the unit of analysis. Per the recommendation of the HCUP, the organization which oversees the NEDS database, sample weights were not used in the multivariable models [16]. We also estimated the proportion of ED visits that were potentially due to attempted self-induced abortion. Because the weighted estimates are nationally representative, we were also able to use published national estimates of abortion incidence [17] to estimate the major incident rate for abortion in the U.S. during the study period. This assumes that the vast majority of major incidents go through an ED evaluation (although we acknowledge that a small percentage do not). For all analyses, we report weighted results unless otherwise specified. Where data were missing, a missing category was retained for all analyses. Statistical significance was set at *P* < 0.05 for all chi-squared tests and adjusted odds ratios (AORs). We used STATA 14 for all analyses.

## Results

Among 42,493,214 unweighted visits among women of reproductive age (15–49) during the study period, 6342 visits had an abortion diagnosis. Among these, 70 visits were determined to be unrelated to abortion based on clinician review and 33 visits were duplicates (all variables were identical other than ID number); these 103 visits were excluded, leaving an analytical sample of 6239 unweighted abortion-related ED visits. We did not include an additional 101 visits with abortion diagnoses for women outside the age range of interest (younger than 15 or older than 49).

The final analytical sample corresponded to 27,941 weighted ED visits for abortion-related reasons among 189,480,685 weighted ED visits among women of reproductive age. Thus abortion-related ED visits represented 0.01% of all ED visits among women of reproductive age during the study period. All subsequent results are weighted.

The average age of the population seeking abortion-related ED care was 26, and 12.9% of visits were by women under 20 (Table 1). Women using Medicaid were most common (45.2% of visits), followed by those using private insurance (31.4%), and those who self-paid (17.1%). The population seeking abortion-related ED care was overwhelmingly of urban residence (91.0%). Low-income zip code residences were overrepresented in the sample; 27.5% of women lived in a zip code with the lowest national income quartile, while 20.5% lived in the highest income quartile. Comorbid conditions including diabetes (1.5%), hypertension (3.2%), and overweight/obese BMI (1.8%) were noted in abortion-related ED visits. Hospitals in the South (35.5%) and West (31.7%) had the largest number of abortion-related ED visits. Most visits were to non-trauma or trauma level III hospitals (62.8%) and most were to hospitals in urban locations (92.3%). Visit numbers remained approximately constant throughout the study period. Average ED costs were $4719, with 8.6% of ED visits costing $10,000 or more.

**Table 1:** Characteristics of abortion-related emergency department visits, 2009–2013 weighted *n* = 27,941

	Weighted *N*	Weighted %	Abortion patients in the U.S., 2014% [15]
Total	27,941	100	100
Patient characteristics			
Age			
15–19	3605	12.9	11.7
20–24	9686	34.7	33.6
25–29	6952	24.9	26.5
30–39	6809	24.4	25.0
40–49	888	0.3	3.1
Primary payer			
Private insurance	8787	31.4	31.3
Medicaid	12,624	45.2	34.6
Medicare	410	1.5	–
Self-pay	4764	17.1	27.6^A^
No charge	188	0.7	–
Other	1049	3.8	6.5^B^
Missing	118	0.4	–
Urban/rural residence			
Urban	25,435	91.0	
Rural	2401	8.6	
Missing	104	0.4	
Zip code-based national income quartile			
First quartile (low)	7686	27.5	65.3^C^
Second quartile	7014	25.1	
Third quartile	7082	25.3	21.7
Fourth quartile (high)	5721	20.5	
Missing	437	1.6	13.0
Comorbidities			
Diabetes	415	1.5	
Hypertension	879	3.1	
Overweight/obese BMI	507	1.8	
Hospital characteristics			
Region			
Northeast	4539	16.2	
Midwest	4627	16.6	
South	9912	35.5	
West	8862	31.7	
Trauma level of hospital			
Level I or II	9429	33.7	
Nontrauma or level III	17,537	62.8	
Not specified	974	3.5	
Urban/rural location of hospital			
Urban	25,796	92.3	
Rural	2145	7.7	
Visit characteristics			
ED visit day			
Weekday	20,640	73.9	
Weekend	7301	26.1	
ED visit season			
Fall	5628	20.1	
Winter	5989	21.4	
Spring	6343	22.7	
Summer	6174	22.1	
Missing	3808	13.6	
Year			
2009	5350	19.1	
2010	5899	21.1	
2011	5448	19.5	
2012	5627	20.1	
2013	5617	20.1	
Total ED charges			
<$1000	2643	9.5	
$1000–$1999	4290	15.4	
$2000–$4999	7188	25.7	
$5000–$9999	3083	11.0	
$10,000+	2407	8.6	
Missing	8330	29.8	

*BMI* body mass index, *ED* Emergency department

^A^Defined as no coverage

^B^Defined as had either insurance through Healthcare.gov or a different type of insurance

^C^Defined as poor (<100% federal poverty level) or low income (<200% federal poverty level). At the time of the study, the national income median was approximately equal to 200% federal poverty level

Half of abortion-related visits received observation care only (50.6%, 95% confidence interval, 95% CI 49.3–51.9%) (Table 2). Nearly a third of visits resulted in a uterine reaspiration or repeat abortion procedure (32.2%, 95% CI 31.0–33.4%). Medications were used in 16.1% of ED visits (95% CI 15.2–17.1%): IV-medications in 13.7% of visits and non-IV medications in 7.4% of visits. The most commonly administered medication was pain medication (10.4%, 95% CI 9.7–11.2%), followed by anti-nausea medication (7.4%, 95% CI 6.8–8.1%) and antibiotics (3.2%, 95% CI 2.8–3.7%). A minority of ED visits involved blood transfusion (5.0%, 95% CI 4.5–5.6%), abortion-related surgery (1.0%, 95% CI 0.7–1.2%), or other treatments (0.3%, 95% CI 0.2–0.5%), and 19.4% (95% CI 18.5–20.5%) of all abortion-related visits resulted in an overnight inpatient stay at the same hospital. Major incidents, treated with blood transfusion, surgery, or overnight inpatient stay, accounted for 20.3% (95% CI 19.3–21.3%) of all visits. Minor incidents accounted for 36.1% (95% CI 34.9–37.4%) of visits.

**Table 2:** Diagnoses and treatments received, weighted *n* = 27,941

	Weighted *N*	Weighted percentage	Weighted percentage 95% confidence interval
Incident type			
Minor incident^A^	10,089	36.1	34.9–37.4
Major incident^B^	5673	20.3	19.3–21.3
No incident	16,087	57.6	56.3–58.8
Could not be determined	426	1.5	1.2–1.9
Treatment received			
Repeat abortion or uterine reaspiration	8994	32.2	31.0–33.4
IV medications	3838	13.7	12.9–14.6
Non-IV medications	2072	7.4	6.8–8.1
Unspecified type medication	232	0.8	0.6–1.1
Type of medication			
Pain	2912	10.4	9.7–11.2
Nausea	2080	7.4	6.8–8.1
Antibiotics	895	3.2	2.8–3.7
Other	1361	4.9	4.3–5.5
Blood transfusion	1397	5.0	4.5–5.6
Surgery	267	1.0	0.7–1.2
Observation care only	14,126	50.6	49.3–51.9
Other treatment^C^	81	0.3	0.1–0.5
Discharge status			
Routine discharge from ED	20,992	75.1	74.0–76.2
Admission to hospital	6136	22.0	20.9–23.0
Transferred to other medical facility	464	1.7	1.4–2.0
Left against medical advice	277	1.0	0.8–1.3
Other or unknown	57	0.2	0.1–0.4
Death	15	0.05	0.02–0.2

*ED* emergency department, *IV* intravenous

^A^Minor incident includes all other incidents that involved an abortion-related diagnosis or treatment, such as those requiring medication or repeat procedure

^B^Major incident includes those requiring overnight inpatient stay, blood transfusion, or surgery

^C^Other treatments include suture of laceration to the cervix, laminaria insertion, and abscess drainage

Three-quarters (75.1%) of abortion-related ED visits resulted in discharge from the ED. Another 21.2% were admitted to the same hospital for abortion-related reasons and 0.8% were admitted for non-abortion-related reasons. For the remaining 3%, the disposition was unknown or they were transferred to another medical facility or left against medical advice. Among all abortion-related ED visits over the 5 years of data (*n* = 27,941), 15 ended in the patient’s death.

Several demographic and hospital factors were significantly associated with major incidents in a multivariable model (Table 3). Women over 30 were more likely than women aged 20–24 to have major incidents (*P* < 0.001), and women using Medicaid (*P* = 0.004) were more likely than women with private insurance to have a major incident. Women who paid out of pocket for the ED visit were less likely to have a major incident than women with private insurance (*P* = 0.001). The presence of any of the three examined comorbid conditions was associated with more than double the odds of a major incident compared to women without those comorbid conditions (*P* < 0.01). ED visits at trauma hospitals were more likely to be for major incidents than those at non-trauma hospitals (*P* < 0.001). ED visits in all regions were significantly more likely to be for a major incident than those in the Midwest (*P* < 0.001). There was a decreasing trend in major incidents over time. Similar significant relationships were found for factors associated with receipt of observation care only, but factors associated with increased likelihood of major incidents were associated with a lower likelihood of requiring observation care only. Two notable exceptions are a significantly higher likelihood of observation care only in the West compared to the Midwest (*P* < 0.001) and the absence of a trend over time for receipt of observation care only.

**Table 3:** Factors associated with major incidents and observation care only, weighted *n* = 27,941

	Major incident	Observation care
	Adjusted odds ratio (95% confidence interval)	Adjusted odds ratio (95% confidence interval)
Age		
15–19	0.94 (0.74–1.21)	1.18 (0.97–1.42)
20–24	Reference	Reference
25–29	1.08 (0.91–1.28)	0.81** (0.71–0.94)
30–39	1.49*** (1.24–1.79)	0.72*** (0.62–0.83)
40–49	1.75** (1.21–2.53)	0.55*** (0.39–0.77)
Primary payer		
Private insurance	Reference	Reference
Medicaid	1.28** (1.08–1.52)	0.83* (0.72–0.96)
Medicare	1.30 (0.79–2.15)	0.94 (0.55–1.62)
Self-pay	0.66** (0.52–0.85)	1.33** (1.12–1.57)
No charge	1.20 (0.39–3.69)	0.73 (0.34–1.59)
Other	1.35 (0.93–1.97)	0.95 (0.68–1.31)
Missing	0.32 (0.06–1.70)	0.85 (0.36–2.00)
Urban/rural residence		
Urban	Reference	Reference
Rural	1.02 (0.59–1.75)	0.86 (0.58–1.28)
Missing	1.67 (0.58–4.75)	0.43 (0.14–1.28)
Zip code-based income quartile		
First quartile	Reference	Reference
Second quartile	1.11 (0.90–1.36)	0.95 (0.82–1.10)
Third quartile	0.93 (0.75–1.15)	0.89 (0.76–1.04)
Fourth quartile	1.04 (0.82–1.32)	0.92 (0.77–1.11)
Missing	0.90 (0.46–1.75)	0.96 (0.54–1.70)
Diabetes indicated		
No	Reference	Reference
Yes	2.47** (1.42–4.31)	0.28*** (0.14–0.54)
Hypertension indicated		
No	Reference	Reference
Yes	3.79*** (2.46–5.83)	0.52** (0.34–0.80)
Overweight/obese BMI indicated		
No	Reference	Reference
Yes	4.63*** (2.65–8.10)	0.18*** (0.10–0.33)
Hospital characteristics		
Region		
Northeast	1.89*** (1.32–2.71)	0.65** (0.48–0.88)
Midwest	Reference	Reference
South	2.11*** (1.53–2.90)	0.69** (0.54–0.86)
West	1.80*** (1.30–2.49)	2.19*** (1.71–2.80)
Trauma level of hospital		
Level I or II	1.52*** (1.23–1.88)	0.81* (0.68–0.98)
Nontrauma or level III	Reference	Reference
Not specified	1.40 (0.88–2.23)	0.86 (0.55–1.35)
Urban or rural location of hospital		
Urban	Reference	Reference
Rural	0.65 (0.36–1.17)	1.20 (0.77–1.86)
Year		
2009	Reference	Reference
2010	0.85 (0.68–1.07)	1.04 (0.85–1.28)
2011	0.77* (0.61–0.98)	1.09 (0.88–1.36)
2012	0.73** (0.57–0.92)	0.95 (0.76–1.19)
2013	0.71** (0.56–0.90)	0.87 (0.70–1.08)

* *P* < 0.05, ** *P* < 0.01, *** *P* < 0.001

*BMI* body mass index

We used published rates of the total number of abortions to calculate the percentage of all abortions seen in an ED that resulted in a major incident. During the 5-year study period, there were an estimated 5,282,500 total abortions in the U.S. [17]. Using this number as the denominator, we estimate the rate of major incidents seen in EDs for abortion in the U.S. is 0.11%, or 108 per 100,000 abortions.

We identified 390 ED visits that represented potential self-induced abortion and accounted for 1.4% of abortion-related ED visits during the study period (95% CI 1.1–1.7%). There were slightly higher rates of potential self-induced abortion in the South (2.0%) than in the Midwest (1.0%), West (1.1%), and Northeast (1.3%) (*P* = 0.05) (Table 4). There were no time trends or other factors associated with self-induced abortion.

**Table 4:** Potential self-induced abortion, by region

	Weighted *N*	Weighted percentage of all abortion-related emergency department visits	Weighted percentage 95% confidence interval	Chi-squared test *P* value
Overall	390	1.4	1.1–1.7	
Region				0.048
Northeast	58	1.3	0.7–2.2	
Midwest	44	1.0	0.5–1.9	
South	195	2.0	1.5–2.6	
West	94	1.1	0.7–1.6	

## Discussion

We found that abortion-related ED visits comprised 0.01% of ED visits among women aged 15–49. In other words, 14 of every 100,000 ED visits among women aged 15–49 were for abortion-related reasons. The majority (51%) of these were visits involving observation care only.

These data also allowed us to estimate the national major incident rate after an abortion. The rate of 0.11% (108 per 100,000) abortion patients is slightly higher than the rate of 0.05% found in a study of first trimester abortion patients in California [18], and slightly lower than the rate of 0.23% found in a study of all abortions covered by California’s Medicaid program [2]. While not all abortion-related incidents lead to an ED visit and thus, are not reflected in this estimate, we believe that the vast majority of major incidents (those involving a blood transfusion, surgery, or hospital admission) are reflected here. Those that would be missed from this analysis are cases that skip the ED and are directly admitted to a hospital or an outpatient surgicenter (usually for scheduled hospitalization) and complications arising from the small proportion (4%) of abortions done in hospitals [17] and are then directly admitted. Thus, the major incident rate may be slightly underestimated.

The major incident rate for abortion (0.1%) is lower than the published rates for pregnancy (1.4%) [19], as well as other common procedures such as colonoscopy (0.2%) [20], wisdom tooth removal (1.0%) [21], and tonsillectomy (1.4%) [22]. Abortion care is, thus, safer than many other unregulated outpatient procedures. Additionally, we found 15 deaths between 2009 and 2013, which is slightly lower than the total number of abortion-related deaths reported in 2009–2012, the most recent years available (*n* = 24) [23].

Notably, the majority of visits involved observation care only, which is consistent with a previous study [2]. Patients experience a range of post-abortion symptoms, including ongoing uterine cramping and bleeding for up to 3 weeks after the abortion. Patients may not realize that this is normal. Some women do not start to bleed until several days after the abortion, while some stop bleeding and then start again. Increased cramping and bleeding could start several days after the abortion. Patients may not be given ample information about what to expect or they may have trouble differentiating normal post-abortion symptoms from signs of a complication. Patient visits to EDs for non-urgent care have the potential to be costly to the health system. Such visits could be due to several reasons and little research has been done on factors that contribute to patients’ decisions to visit an ED after abortion. EDs offer 24-h access to care compared to abortion facilities, which have relatively limited hours and may require an appointment [24]. The long distances that many women across the U.S. must travel to reach an abortion provider may make return visits for follow-up too arduous [25, 26]. Indeed, research found that patients who travel longer distances to reach an abortion provider are more likely to visit an ED for follow-up care or to manage subsequent symptoms and less likely to return to the original abortion provider [27]. We note that patients presenting to the ED were disproportionately of urban residence (91% in the sample compared to 81% of the U.S. population) [28]. This is likely explained in that 91% of abortion patients live in urban areas [29] and 92% of EDs in the sample were in urban areas, attracting mainly local residents who may find them convenient geographically.

Post-abortion visits to the ED may also be driven by stigma, worry, or distrust of abortion providers. A perception that abortion is unsafe [30–32] may lead women to worry about mild symptoms, such as cramping and bleeding, even though they are an expected result of abortion. Such perceptions may stem from abortion portrayals in the media and popular culture. A study of abortion-related storylines in fictional American television shows found a major incident rate of 34% [33], over 34,000% greater than the real-life major incident rate of 0.1% found here. The long-term consequences of these fictional abortions were much more likely to be severe, including frequent depictions of negative mental health sequelae, infertility, and even death.

Women using Medicaid had higher odds of major incidents than those not using Medicaid and lower odds of observation care. In this context, insurance type may be a proxy for socioeconomic status, as women requiring Medicaid are low income and as a result, face a multitude of barriers to accessing health care and are known to have poorer health status, including multiple chronic conditions, than women with private insurance. Women who were self-pay were less likely to have major incidents and more likely to receive observation care only, suggesting that patients without healthcare coverage may not have been given treatments to reduce patient costs.

We found that the pre-existing chronic conditions that have previously been suspected to be associated with major abortion-related incidents were indeed associated with a significantly higher rate of those incidents in this sample. While there is limited previous research on the impact of chronic health conditions on the risk of abortion complications, it is well established that women with chronic conditions are more likely to have pregnancy-related complications [34]. Our findings are consistent with provider guidance that suggests women with multiple chronic medical conditions may be at increased risk [35], but conflicting with previous studies that find that obesity and chronic health conditions confer no increased risk among women having abortions [11–14]. This increased risk might be explained in that women who had overnight inpatient stays (one of the categories of a major incident) were more likely to have their obesity or other chronic diseases documented in their charts than those who did not have an overnight stay.

We found some cases of potential self-induced abortions using such means as poisoning or other methods of self-harm. While self-induced abortions are safe when appropriate dosages of mifepristone and misoprostol or misoprostol alone are used, other methods are hazardous, as evidenced by ED visits. Rates were highest in the South, which is known to have the most barriers to abortion access, including the fewest providers [17, 26], and the most state-level restrictions. States that are hostile to abortion may see more ED visits due to self-induced abortion than non-hostile states, potentially due to stigma, protesters, and other barriers to in-clinic abortion.

This study included a large, nationally representative sample of ED visits, allowing us to draw national- and regional-level conclusions about abortion safety. However, using billing codes to understand the nature of the ED visit can be imprecise and incomplete. The estimates produced here may be conservative if patients did not report having had an abortion due to fear of stigmatization or if relevant diagnosis and procedure codes were not reported or were systematically misreported. The lack of full clinical data to determine abortion relatedness could cause errors. For example, the visits in this study could include cases of miscarriage. Likewise, this study may miss abortion-related incidents that were inaccurately coded as a miscarriage.

## Conclusions

These new findings can inform policy debates regarding abortion regulation in the U.S. Regulations on abortion provider or facility relationships to hospitals or EDs should be considered in light of their relative impact on improving women’s health. Because abortion-related ED visits comprise a very small proportion of women’s ED visits, and the rate of major incidents is very low, regulations on abortion are unlikely to have any impact on women’s health outcomes. Many abortion-related ED visits are for observation only and may not be indicated or could be managed at a less costly level of care. Perceptions that abortion is unsafe are not based on evidence.

## Abbreviations

AOR: Adjusted odds ratio
BMI: Body mass index
CI: Confidence interval
CPT: Current Procedural Terminology
ED: Emergency department
HCPCS: Healthcare Common Procedure Coding System
HCUP: Healthcare Cost and Utilization Project
ICD-9: International Classification of Diseases, 9th Revision
IV: Intravenous
NEDS: Nationwide Emergency Department Sample
PAIRS: Procedural Abortion Incident Reporting and Surveillance
U.S.: United States
